# Supplementary material for: Early childhood caries intervention in Aboriginal Australian children: Follow-up at child age 9 years
Source: PLoS One. 2025 Sep 3;20(9):e0317024. doi: 10.1371/journal.pone.0317024 (PMC12407408; doi:10.1371/journal.pone.0317024)
Supplement: S1 Table — (DOCX) [file pone.0317024.s001.docx]

**Supplementary**

S1Table: Baseline mother-child pairs characteristics and dental caries among Indigenous Australians at 9 years follow-up

|  | Deciduous teeth (Mean, 95% CI) | | | | Permanent teeth (Mean, 95% CI) | | | |
| --- | --- | --- | --- | --- | --- | --- | --- | --- |
|  | dt | ft | mt | dmft | DT | FT | MT | DMFT |
| **Total** | 0.66 (0.43-0.84) | 0.73 (0.57-0.89) | 2.23 (1.67-2.39) | 3.41 (2.95-3.87) | 0.19 (0.12-0.26) | 0.10 (0.05-0.15) | 0.02 (0.00-0.04) | 0.31 (0.22-0.41) |
| **Intervention group** |  |  |  |  |  |  |  |  |
| DI | 0.71 (0.46-0.96) | 0.81 (0.57-1.06) | 2.09 (1.57-2.60) | 3.61 (2.92-4.30) | 0.17 (0.06-0.28) | 0.10 (0.03-0.18) | 0.03 (0.00-0.07) | 0.30 (0.16-0.44) |
| II | 0.62 (0.38-0.85) | 0.64 (0.42-0.86) | 1.97 (1.46-2.47) | 3.20 (2.59-3.82) | 0.21 (0.12-0.30) | 0.10 (0.04-0.17) | 0.02 (0.00-0.04) | 0.33 (0.20-0.45) |
| **Mothers’ characteristics** |  |  |  |  |  |  |  |  |
| **Maternal age** |  |  |  |  |  |  |  |  |
| 14-24 | 0.83 (0.57-1.10) | 0.67 (0.47-0.91) | 2.13 (1.59-2.67) | 3.64 (2.96-4.32) | 0.21 (0.10-0.32) | 0.11 (0.03-0.19) | 0.02 (0.00-0.05) | 0.34 (0.20-0.48) |
| 25+ | 0.47 (0.26-0.67) | 0.78 (0.53-1.02) | 1.91 (1.45-2.37) | 3.14 (2.53-3.76) | 0.16 (0.08-0.24) | 0.09 (0.04-0.15) | 0.03 (0.00-0.06) | 0.28 (0.17-0.39) |
| **Education** |  |  |  |  |  |  |  |  |
| ≤12 years | 0.74 (0.53-0.95) | 0.77 (0.57-0.97) | 2.33 (1.85-2.81) | 3.83 (3.23-4.43) | 0.23 (0.16-0.33) | 0.11 (0.04-0.17) | 0.02 (0.00-0.04) | 0.37 (0.24-0.49) |
| >12 years | 0.47 (0.17-0.76) | 0.64 (0.34-0.93) | 1.28 (0.87-1.69) | 2.37 (1.76-2.98) | 0.09 (0.02-0.14) | 0.09 (0.02-0.16) | 0.03 (0.00-0.08) | 0.21 (0.09-0.32) |
| **Source of Income** |  |  |  |  |  |  |  |  |
| Centrelink | 0.73 (0.53-0.93) | 0.71 (0.52-0.90) | 2.13 (1.72-2.55) | 3.56 (3.03-4.08) | 0.20 (0.12-0.29) | 0.09 (0.04-0.15) | 0.01 (0.00-0.03) | 0.31(0.20-0.41) |
| Job | 0.26 (0.01-0.50) | 0.86 (0.47-1.25) | 1.53 (0.90-2.17) | 2.66 (1.68-3.63) | 0.13 (0.03-0.24) | 0.16 (0.03-0.28) | 0.07 (0.00-0.17) | 0.36 (0.16-0.54) |
| **Residential location** |  |  |  |  |  |  |  |  |
| Non-metropolitan | 0.85 (0.61-1.10) | 0.84 (0.62-1.06) | 2.31 (1.83-2.79) | 3.99 (3.39-4.60) | 0.21 (0.11-0.31) | 0.12 (0.05-0.19) | 0.01 (0.00-0.02) | 0.34 (0.21-0.47) |
| Metropolitan | 0.33 (0.15-0.52) | 0.57 (0.32-0.81) | 1.59 (1.04-2.14) | 2.48 (1.78-3.18) | 0.15 (0.06-0.24) | 0.08 (0.02-0.13) | 0.05 (0.00-0.11) | 0.28 (0.16-0.40) |
| **Smoking status** |  |  |  |  |  |  |  |  |
| Current | 0.64 (0.40-0.89) | 0.74 (0.49-0.99) | 2.30 (1.70-2.89) | 3.67 (2.94-4.41) | 0.23 (0.15-0.34) | 0.12 (0.06-0.19) | 0.02 (0.00-0.04) | 0.37 (0.29-0.51) |
| Former | 0.58 (0.27-0.89) | 0.53 (0.30-0.76) | 1.62 (1.04-2.19) | 2.71 (1.99-3.42) | 0.08 (0.01-0.13) | 0.05 (0.00-0.10) | 0.05 (0.00-0.12) | 0.17 (0.06-0.23) |
| Never | 0.78 (0.37-1.20) | 0.99 (0.57-1.40) | 2.02 (1.31-2.74) | 3.80 (2.78-4.81) | 0.25 (0.06-0.44) | 0.13 (0.00-0.28) | 0.02 (0.00-0.05) | 0.39 (0.16-0.63) |
| **Alcohol status** |  |  |  |  |  |  |  |  |
| Current | 0.89 (0.27-1.50) | 0.37 (0.06-0.68) | 2.03 (0.48-3.58) | 3.29 (1.43-5.14) | 0.20 (0.00-0.49) | 0.05 (0.00-0.15) | 0.00 (0.00-0.00) | 0.25 (0.00-0.54) |
| Used | 0.55 (0.37-0.73) | 0.82 (0.62-1.02) | 1.99 (1.61-2.37) | 3.34 (2.84-3.84) | 0.21 (0.12-0.29) | 0.10 (0.04-0.15) | 0.03 (0.00-0.06) | 0.34 (0.23-0.44) |
| Never | 1.41 (0.53-2.28) | 0.56 (0.12-1.00) | 2.69 (0.96-4.42) | 4.66 (2.63-6.68) | 0.12 (0.00-0.26) | 0.20 (0.00-0.41) | 0.00 (0.00-0.00) | 0.32 (0.06-0.57) |
| **Children’s characteristics** |  |  |  |  |  |  |  |  |
| **Sex** |  |  |  |  |  |  |  |  |
| Male | 0.69 (0.44-0.94) | 0.84 (0.59-1.08) | 2.09 (1.56-2.62) | 3.60 (2.92-4.27) | 0.16 (0.08-0.24) | 0.07 (0.02-0.12) | 0.01 (0.00-0.03) | 0.25 (0.14-0.35) |
| Female | 0.63 (0.39-0.87) | 0.61 (0.39-0.84) | 1.98 (1.48-2.49) | 3.22 (2.59-3.86) | 0.23 (0.11-0.36) | 0.14 (0.04-0.25) | 0.04 (0.00-0.08) | 0.41 (0.24-0.58) |
| **Gestation** |  |  |  |  |  |  |  |  |
| Preterm | 1.04 (0.08-2.01) | 0.70 (0.00-1.46) | 1.64 (0.00-3.30) | 3.30 (1.08-5.53) | 0.00 (0.00-0.00) | 0.15 (0.00-0.38) | 0.00 (0.00-0.00) | 0.15 (0.00-0.38) |
| Normal | 0.60 (0.41-0.78) | 0.75 (0.56-0.93) | 2.00 (1.59-2.40) | 3.45 (2.83-3.86) | 0.21 (0.13-0.29) | 0.10 (0.04-0.15) | 0.03 (0.00-0.06) | 0.34 (0.23-0.45) |
| **Baby birth weight** |  |  |  |  |  |  |  |  |
| Low | 0.62 (0.00-1.23) | 0.38 (0.04-0.72) | 1.16 (0.09-2.23) | 2.12 (0.74-3.49) | 0.13 (0.00-0.33) | 0.13 (0.00-0.33) | 0.00 (0.00-0.00) | 0.27 (0.00-0.60) |
| Normal | 0.47 (0.30-0.64) | 0.77 (0.57-0.97) | 2.06 (1.61-2.50) | 3.30 (2.73-3.87) | 0.19 (0.11-0.28) | 0.08 (0.03-0.12) | 0.03 (0.00-0.06) | 0.30 (0.19-0.40) |
| **Breast feeding** |  |  |  |  |  |  |  |  |
| No | 0.69 (0.44-0.94) | 0.69 (0.49-0.89) | 2.11 (1.60-2.62) | 3.48 (2.83-4.13) | 0.22 (0.12-0.31) | 0.13 (0.04-0.21) | 0.01 (0.00-0.04) | 0.36 (0.22-0.49) |
| Yes | 0.60 (0.35-0.86) | 0.83 (0.52-1.14) | 1.99 (1.41-2.57) | 3.42 (2.68-4.17) | 0.16 (0.04-0.28) | 0.07 (0.02-0.12) | 0.04 (0.00-0.09) | 0.27 (0.13-0.41) |
| **Sweet food consumption** |  |  |  |  |  |  |  |  |
| > 15% | 0.82 (0.02-1.63) | 0.41 (0.13-0.70) | 1.53 (0.42-2.64) | 2.76 (1.46-4.07) | 0.20 (0.00-0.49) | 0.10 (0.00-0.24) | 0.10 (0.00-0.24) | 0.40 (0.02-0.78) |
| 11%-15% | 0.62 (0.36-0.89) | 0.76 (0.50-1.03) | 2.17 (1.56-2.77) | 3.55 (2.81-4.30) | 0.22 (0.09-0.35) | 0.10 (0.04-0.17) | 0.00 (0.00-0.00) | 0.32 (0.17-0.48) |
| 5%-10% | 0.65 (0.25-1.05) | 1.17 (0.62-1.72) | 2.34 (1.31-3.37) | 4.12 (2.74-5.49) | 0.17 (0.01-0.33) | 0.12 (0.00-0.25) | 0.05 (0.00-0.12) | 0.34 (0.12-0.56) |
| < 5% | 1.35 (0.09-2.61) | 0.88 (0.00-1.86) | 2.00 (0.25-3.75) | 4.24 (1.55-6.92) | 0.10 (0.00-0.33) | 0.00 (0.00-0.00) | 0.00 (0.00-0.00) | 0.10 (0.00-0.32) |
| **Tooth brushing** |  |  |  |  |  |  |  |  |
| < 2/day | 0.78 (0.53-1.04) | 0.75 (0.54-0.97) | 2.05 (1.56-2.53) | 3.58 (2.98-4.18) | 0.21 (0.11-0.32) | 0.11 (0.04-0.18) | 0.04 (0.00-0.08) | 0.36 (0.23-0.50) |
| ≥ 2/day | 0.35 (0.17-0.52) | 0.96 (0.54-1.39) | 2.00 (1.25-2.75) | 3.29 (2.23-4.36) | 0.14 (0.05-0.23) | 0.07 (0.00-0.15) | 0.00 (0.00-0.00) | 0.21 (0.09-0.33) |

Notes: II: Immediate intervention, DI: delayed intervention.
