# Supplementary material for: Early childhood caries intervention in Aboriginal Australian children: Follow-up at child age 9 years
Source: PLoS One. 2025 Sep 3;20(9):e0317024. doi: 10.1371/journal.pone.0317024 (PMC12407408; doi:10.1371/journal.pone.0317024)
Supplement: S2 Table — (DOCX) [file pone.0317024.s002.docx]

S2 Table: Models for the mean number of dt at 9 years follow-up (RR, 95% CI)

|  | Model 1 | Model 2 | Model 3 | Model 4 |
| --- | --- | --- | --- | --- |
|  | RR (95% CI) | RR (95% CI) | RR (95% CI) | RR (95% CI) |
| **Intervention group** |  |  |  |  |
| DI | 1.15 (0.89-1.49) | 1.20 (0.91-1.57) | 1.14 (0.80-1.62) | 1.15 (0.81-1.65) |
| II | ref | ref | ref | ref |
| **Mothers’ characteristics at baseline** | |  |  |  |
| **Maternal age** |  |  |  |  |
| 14-24 | **1.79 (1.36-2.34) | **1.84 (1.35-2.50) |  | ***2.76 (1.76-4.31) |
| 25+ | ref | ref |  | ref |
| **Education Level** |  |  |  |  |
| ≤12 years | **1.59 (1.16-2.18) | 1.34 (0.94-1.89) |  | **1.49 (1.19-1.76) |
| >12 years | ref | ref |  | ref |
| **Source of Income** |  |  |  |  |
| Centrelink | **2.82 (1.67-4.75) | 1.84 (1.06-3.20) |  | 1.26 (0.70-2.27) |
| Job | ref | ref |  | ref |
| **Residential location** |  |  |  |  |
| Non-metropolitan | **2.56 (1.84-3.57) | **2.88 (2.00-4.15) |  | **1.67 (1.38-2.00) |
| Metropolitan | ref | ref |  | ref |
| **Smoking status** |  |  |  |  |
| Current | 0.82 (0.61-1.12) | 0.91 (0.65-1.27) |  | 0.99 (0.63-1.56) |
| Former | 0.74 (0.51-1.07) | 1.03 (0.69-1.54) |  | 0.69 (0.41-1.17) |
| Never | ref | ref |  | ref |
| **Alcohol status** |  |  |  |  |
| Current | 0.63 (0.40-1.00) | *0.57 (0.38-0.85) |  | 1.94 (0.78-4.84) |
| Used | *0.39 (0.27-0.54) | 1.11 (0.67-1.83) |  | 2.41 (0.84-6.91) |
| Never | ref | ref |  | ref |
| **Children’s characteristics** |  |  |  |  |
| **Sex** |  |  |  |  |
| Male | 1.10 (0.85-1.42) |  | 1.15 (0.80-1.65) | 1.29 (0.88-1.89) |
| Female | ref |  | ref | ref |
| **Gestation** |  |  |  |  |
| Preterm | **1.74 (1.14-2.67) |  | 1.60 (0.49-2.03) | **1.71 (1.18-2.48) |
| Normal | ref |  | ref | ref |
| **Baby birth weight** |  |  |  |  |
| Low | 1.31 (0.77-2.20) |  | 0.76 (0.43-1.35) | 1.32 (0.92-1.89) |
| Normal | ref |  | ref | ref |
| **Breast feeding** |  |  |  |  |
| No | 1.14 (0.87-1.50) |  | 1.37 (0.95-1.98) | 1.18 (0.80-1.76) |
| Yes | ref |  | ref | ref |
| **Free sugar consumption of total energy intake** | |  |  |  |
| > 15% | *1.64 (1.01-2.85) |  | 1.15 (0.53-2.48) | 1.26 (0.78-2.04) |
| 11%-15% | 0.79 (0.49-1.25) |  | 1.00 (0.56-1.78) | 0.78 (0.41-1.47) |
| 5%-10% | 0.76 (0.49-1.16) |  | 0.48 (0.27-0.82) | 0.62 (0.29-1.38) |
| < 5% | ref |  | ref | ref |
| **Tooth brushing** |  |  |  |  |
| < 2/day | **2.21 (1.49-3.28) |  | **2.93 (1.59-5.41) | **2.23 (1.19-4.14) |
| ≥ 2/day | ref |  | ref | ref |

Notes: RR: risk ratio, II: Immediate intervention, DI: delayed intervention; *P<0.05, **P<0.01, ***P<0.001.
