# Supplementary material for: Early childhood caries intervention in Aboriginal Australian children: Follow-up at child age 9 years
Source: PLoS One. 2025 Sep 3;20(9):e0317024. doi: 10.1371/journal.pone.0317024 (PMC12407408; doi:10.1371/journal.pone.0317024)
Supplement: S3 Table — (DOCX) [file pone.0317024.s003.docx]

S3 Table: Models for the mean number of ft at 9 years follow-up (RR, 95% CI)

|  | Model 1 | Model 2 | Model 3 | Model 4 |
| --- | --- | --- | --- | --- |
|  | RR (95% CI) | RR (95% CI) | RR (95% CI) | RR (95% CI) |
| **Intervention group** |  |  |  |  |
| DI | *1.26 (1.01-1.61) | *1.31(1.02-1.68) | 1.23 (0.93-1.64) | 1.32 (0.98-1.76) |
| II | ref | ref | ref | ref |
| **Mothers’ characteristics at baseline** | |  |  |  |
| **Maternal age** |  |  |  |  |
| 14-24 | 0.89 (0.70-1.13) | 0.81 (0.62-1.05) |  | 0.82 (0.60-1.12) |
| 25+ | ref | ref |  | ref |
| **Education** |  |  |  |  |
| ≤12 years | 1.21 (0.92-1.59) | *1.36 (1.01-1.84) |  | **1.73 (1.22-2.46) |
| >12 years | ref | ref |  | ref |
| **Source of Income** |  |  |  |  |
| Centrelink | 0.83 (0.61-1.12) | 0.84 (0.59-1.19) |  | 0.78 (0.53-1.13) |
| Job | ref | ref |  | ref |
| **Residential location** |  |  |  |  |
| Non-metropolitan | **1.48 (1.13-1.94) | **1.57 (1.19-2.07) |  | ***1.88 (1.36-2.62) |
| Metropolitan | ref | ref |  | ref |
| **Smoking status** |  |  |  |  |
| Current | *0.75 (0.57-0.99) | *0.69 (0.51-0.94) |  | *0.51 (0.35-0.74) |
| Former | *0.54 (0.37-0.77) | *0.49 (0.33-0.70) |  | *0.52 (0.35-0.76) |
| Never | ref | ref |  | ref |
| **Alcohol status** |  |  |  |  |
| Current | 0.66 (0.32-1.35) | **2.23 (1.27-3.93) |  | 1.54 (0.80-2.97) |
| Used | 1.45 (0.90-2.36) | 1.27 (0.62-2.65) |  | 0.79 (0.33-1.86) |
| Never | ref | ref |  | ref |
| **Children’s characteristics** | |  |  |  |
| **Sex** |  |  |  |  |
| Male | *1.36 (1.06-1.75) |  | 1.23 (0.92-1.64) | 0.79 (0.34-1.86) |
| Female | ref |  | ref | ref |
| **Gestation** |  |  |  |  |
| Preterm | 0.93 (0.56-1.54) |  | 0.70 (0.39-1.24) | 1.29 (0.96-1.74) |
| Normal | ref |  | ref | ref |
| **Baby birth weight** |  |  |  |  |
| Low | 0.50 (0.26-0.94) |  | **2.37 (1.21-4.64) | ***2.56 (1.21-5.40) |
| Normal | ref |  | ref | ref |
| **Breast feeding** |  |  |  |  |
| No | 0.83 (0.65-1.06) |  | 0.80 (0.60-1.06) | 0.88 (0.65-1.19) |
| Yes | ref |  | ref | ref |
| **Free sugar consumption of total energy intake** | |  |  |  |
| > 15% | *0.47 (0.23-0.97) |  | *0.30 (0.14-0.67) | *0.37 (0.15-0.86) |
| 11%-15% | 0.86 (0.50-1.48) |  | 0.68 (0.39-1.19) | 0.94 (0.53-1.68) |
| 5%-10% | 1.32 (0.76-2.31) |  | 0.94 (0.52-1.69) | 1.46 (0.79-2.69) |
| < 5% | ref |  | ref | ref |
| **Tooth brushing** |  |  |  |  |
| < 2/day | 0.78 (0.60-1.02) |  | 1.03 (0.73-1.45) | 0.95 (0.66-1.36) |
| ≥ 2/day | ref |  | ref | ref |

Notes: RR: risk ratio, II: Immediate intervention, DI: delayed intervention; *P<0.05, **P<0.01, ***P<0.001.
