# Supplementary material for: Early childhood caries intervention in Aboriginal Australian children: Follow-up at child age 9 years
Source: PLoS One. 2025 Sep 3;20(9):e0317024. doi: 10.1371/journal.pone.0317024 (PMC12407408; doi:10.1371/journal.pone.0317024)
Supplement: S4 Table — (DOCX) [file pone.0317024.s004.docx]

S4 Table: Models for the mean number of DMFT at 9 years follow-up (RR, 95% CI)

|  | Model 1 | Model 2 | Model 3 | Model 4 |
| --- | --- | --- | --- | --- |
|  | RR (95% CI) | RR (95% CI) | RR (95% CI) | RR (95% CI) |
| **Intervention group** |  |  |  |  |
| DI | 0.93 (0.60-1.44) | 0.96 (0.61-1.50) | 0.65 (0.38-1.16) | 0.63 (0.36-1.10) |
| II | ref | ref | ref | ref |
| **Mothers’ characteristics at baseline** | |  |  |  |
| **Maternal age** |  |  |  |  |
| 14-24 | 1.21 (0.77-1.88) | 1.19 (0.74-1.91) |  | 1.20 (0.66-2.16) |
| 25+ | ref | ref |  |  |
| **Education** |  |  |  |  |
| ≤12 years | **1.78 (1.03-3.08) | **1.79 (1.02-3.23) |  | *1.87 (1.00-3.65) |
| >12 years | ref | ref |  | ref |
| **Source of Income** |  |  |  |  |
| Centrelink | 0.87 (0.50-1.50) | 0.67 (0.35-1.28) |  | 0.66 (0.33-1.31) |
| Job | ref | ref |  | ref |
| **Residential location** |  |  |  |  |
| Non-metropolitan | 1.21 (0.76-1.93) | 1.26 (0.78-2.04) |  | 1.14 (0.65-2.01) |
| Metropolitan | ref | ref |  | ref |
| **Smoking status** |  |  |  |  |
| Current | 0.95 (0.57-1.55) | 0.96 (0.54-1.68) |  | 0.89 (0.46-1.72) |
| Former | **0.44 (0.22-0.91) | **0.46 (0.22-0.95) |  | 0.53 (0.24-1.16) |
| Never | ref | ref |  | ref |
| **Alcohol status** |  |  |  |  |
| Current | 0.78 (0.26-2.39) | 1.41 (0.56-3.58) |  | 0.76 (0.25-2.29) |
| Used | 1.05 (0.50-2.18) | 1.07 (0.33-3.41) |  | 0.90 (0.24-3.42) |
| Never | ref | ref |  | ref |
| **Children’s characteristics** |  |  |  |  |
| **Sex** |  |  |  |  |
| Male | *0.59 (0.38-0.93) |  | 0.66 (0.39-1.13) | 0.70 (0.40-1.21) |
| Female | ref |  | ref | ref |
| **Gestation** |  |  |  |  |
| Preterm | 0.45 (0.11-1.86) |  | 3.94 (0.52-30.03) | 4.55 (0.60-34.75) |
| Normal | ref |  | ref | ref |
| **Baby birth weight** |  |  |  |  |
| Low | 0.89 (0.32-2.47) |  | 0.84 (0.29-2.39) | 0.72 (0.25-2.13) |
| Normal | ref |  | ref | ref |
| **Breast feeding** |  |  |  |  |
| No | 1.33 (0.83-2.13) |  | 1.01 (0.59-1.73) | 0.92 (0.53-1.60) |
| Yes | ref |  | ref | ref |
| **Free sugar consumption of total energy intake** | |  |  |  |
| > 15% | 4.00 (0.50-32.00) |  | 2.67 (0.32-22.2) | 3.40 (0.39-29.68) |
| 11%-15% | 3.24 (0.44-23.65) |  | 2.09 (0.28-15.65) | 2.47 (0.32-19.29) |
| 5%-10% | 3.41 (0.45-26.00) |  | 2.03 (0.24-16.93) | 2.21 (0.25-19.25) |
| < 5% | ref |  | ref | ref |
| **Tooth brushing** |  |  |  |  |
| < 2/day | 1.74 (0.93-3.26) |  | 1.77 (0.77-4.09) | 1.76 (0.72-4.30) |
| ≥ 2/day | ref |  | ref | ref |

Notes: RR: risk ratio, II: Immediate intervention, DI: delayed intervention; *P<0.05, **P<0.01, ***P<0.001.
