# Supplementary material for: Early childhood caries intervention in Aboriginal Australian children: Follow-up at child age 9 years
Source: PLoS One. 2025 Sep 3;20(9):e0317024. doi: 10.1371/journal.pone.0317024 (PMC12407408; doi:10.1371/journal.pone.0317024)
Supplement: S5 Table — (DOCX) [file pone.0317024.s005.docx]

S5 Table: Models for the mean number of DT at 9 years follow-up (RR, 95% CI)

|  | Model 1 | Model 2 | Model 3 | Model 4 |
| --- | --- | --- | --- | --- |
|  | RR (95% CI) | RR (95% CI) | RR (95% CI) | RR (95% CI) |
| **Intervention group** |  |  |  |  |
| DI | 0.80 (0.45-1.41) | 0.79 (0.44-1.41) | 0.51 (0.26-1.01) | 0.43 (0.21-0.88) |
| II | ref | ref | ref | ref |
| **Mothers’ characteristics at baseline** | |  |  |  |
| **Maternal age** |  |  |  |  |
| 14-24 | 1.29 (0.73-2.30) | 1.18 (0.64-2.19) |  | 1.70 (0.78-3.72) |
| 25+ | ref | ref |  | ref |
| **Education** |  |  |  |  |
| ≤12 years | **2.61 (1.17-5.82) | *2.29 (1.01-5.38) |  | *2.19 (1.02-5.57) |
| >12 years | ref | ref |  | ref |
| **Source of Income** |  |  |  |  |
| Centrelink | 1.52 (0.65-3.58) | 1.23 (0.47-3.22) |  | 1.38 (0.51-3.69) |
| Job | ref | ref |  | ref |
| **Residential location** |  |  |  |  |
| Non-metropolitan | 1.41 (0.76-2.63) | 1.37 (0.72-2.58) |  | 1.34 (0.66-2.72) |
| Metropolitan | ref | ref |  | ref |
| **Smoking status** |  |  |  |  |
| Current | 0.94 (0.50-1.76) | 0.70 (0.35-1.40) |  | 0.57 (0.26-1.22) |
| Former | *0.32 (0.12-0.89) | *0.28 (0.10-0.79) |  | *0.23 (0.07-0.72) |
| Never | ref | ref |  | ref |
| **Alcohol status** |  |  |  |  |
| Current | 1.67 (0.37-7.44) | 1.27 (0.44-3.68) |  | 0.48 (0.15-1.58) |
| Used | 1.71 (0.53-5.52) | 0.50 (0.11-2.34) |  | 0.31 (0.06-1.57) |
| Never | ref | ref |  | ref |
| **Children’s characteristics** | |  |  |  |
| **Sex** |  |  |  |  |
| Male | 0.68 (0.39-1.20) |  | 0.71 (0.37-1.38) | 0.78 (0.40-1.53) |
| Female | ref |  | ref | ref |
| **Gestation** |  |  |  |  |
| Preterm | 0.00 (0.00-0.00) |  | 0.00 (0.00-0.00) | 0.00 (0.00-0.00) |
| Normal | ref |  | ref | ref |
| **Baby birth weight** |  |  |  |  |
| Low | 0.69 (0.17-2.87) |  | 0.99 (0.23-4.14) | 1.12 (0.25-4.94) |
| Normal | ref |  | ref | ref |
| **Breast feeding** |  |  |  |  |
| No | 1.35 (0.73-2.49) |  | 1.15 (0.59-2.24) | 0.93 (0.46-1.88) |
| Yes | ref |  | ref | ref |
| **Free sugar consumption of total energy intake** | |  |  |  |
| > 15% | 2.00 (0.22-17.89) |  | 1.79 (0.19-16.80) | 2.19 (0.24-20.3) |
| 11%-15% | 2.19 (0.30-16.22) |  | 1.46 (0.19-10.96) | 1.92 (0.25-14.86) |
| 5%-10% | 1.71 (0.21-13.88) |  | 1.25 (0.14-11.53) | 1.16 (0.12-11.29) |
| < 5% | ref |  | ref | ref |
| **Tooth brushing** |  |  |  |  |
| < 2/day | 1.53 (0.70-3.33) |  | 1.26 (0.49-3.24) | 0.90 (0.29-2.79) |
| ≥ 2/day | ref |  | ref | ref |

Notes: RR: risk ratio, II: Immediate intervention, DI: delayed intervention; *P<0.05, **P<0.01, ***P<0.001.
