# Supplementary material for: Early childhood caries intervention in Aboriginal Australian children: Follow-up at child age 9 years
Source: PLoS One. 2025 Sep 3;20(9):e0317024. doi: 10.1371/journal.pone.0317024 (PMC12407408; doi:10.1371/journal.pone.0317024)
Supplement: S6 Table — (DOCX) [file pone.0317024.s006.docx]

S6 Table: Models for the mean number of FT at 9 years follow-up (RR, 95% CI)

|  | Model 1 | Model 2 | Model 3 | Model 4 |
| --- | --- | --- | --- | --- |
|  | RR (95% CI) | RR (95% CI) | RR (95% CI) | RR (95% CI) |
| **Intervention group** |  |  |  |  |
| DI | 1.02 (0.47-2.21) | 1.13 (0.51-2.46) | 0.74 (0.25-2.17) | 0.88 (0.28-2.81) |
| II | ref | ref | ref | ref |
| **Mothers’ characteristics at baseline** | |  |  |  |
| **Maternal age** |  |  |  |  |
| 14-24 | 1.16 (0.53-2.52) | 1.20 (0.52-2.78) |  | 0.52 (0.16-1.73) |
| 25+ | ref | ref |  | ref |
| **Education** |  |  |  |  |
| ≤12 years | 1.21 (0.51-2.88) | 1.31 (0.51-3.33) |  | 1.53 (0.47-5.03) |
| >12 years | ref | ref |  | ref |
| **Source of Income** |  |  |  |  |
| Centrelink | 0.59 (0.25-1.40) | 0.38 (0.13-1.14) |  | *0.21 (0.06-0.81) |
| Job | ref | ref |  | ref |
| **Residential location** |  |  |  |  |
| Non-metropolitan | 1.58 (0.66-3.75) | 1.80 (0.74-4.41) |  | 1.48 (0.46-4.71) |
| Metropolitan | ref | ref |  | ref |
| **Smoking status** |  |  |  |  |
| Current | 0.95 (0.40-2.23) | 1.47 (0.54-3.98) |  | 2.95 (0.67-13.07) |
| Former | 0.36 (0.10-1.37) | 0.48 (0.12-1.89) |  | 1.27 (0.23-6.95) |
| Never | ref | ref |  | ref |
| **Alcohol status** |  |  |  |  |
| Current | 0.25 (0.03-2.14) | 1.86 (0.24-14.35) |  | 0.00 (0.00-0.00) |
| Used | 0.50 (0.19-1.33) | 3.23 (0.35-29.67) |  | 0.00 (0.00-0.00) |
| Never | ref | ref |  | ref |
| **Children’s characteristics** | |  |  |  |
| **Sex** |  |  |  |  |
| Male | 0.50 (0.23-1.11) |  | 0.55 (0.19-1.62) | 0.72 (0.22-2.33) |
| Female | ref |  | ref | ref |
| **Gestation** |  |  |  |  |
| Preterm | 1.57 (0.37-6.71) |  | 1.21 (0.13-11.2) | 1.36 (0.14-13.24) |
| Normal | ref |  | ref | ref |
| **Baby birth weight** |  |  |  |  |
| Low | 1.72 (0.39-7.58) |  | 0.46 (0.09-2.38) | 0.21 (0.04-1.16) |
| Normal | ref |  | ref | ref |
| **Breast feeding** |  |  |  |  |
| No | 1.81 (0.75-4.37) |  | 1.16 (0.39-3.42) | 1.07 (0.33-3.39) |
| Yes | ref |  | ref | ref |
| **Free sugar consumption of total energy intake** | |  |  |  |
| > 15% | *0.04 (0.00-0.07) |  | **0.09 (0.06-0.12) | **0.09 (0.06-0.12) |
| 11%-15% | **0.05 (0.01-0.06) |  | **0.02 (0.00-0.08) | **0.02 (0.00-0.06) |
| 5%-10% | - |  | - | - |
| < 5% | ref |  | ref | ref |
| **Tooth brushing** |  |  |  |  |
| < 2/day | 1.58 (0.53-4.72) |  | 4.25 (0.50-36.11) | 3.57 (0.32-39.60) |
| ≥ 2/day | ref |  | ref | ref |

Notes: RR: risk ratio, II: Immediate intervention, DI: delayed intervention; *P<0.05, **P<0.01, ***P<0.001.
