# Supplementary material for: Early childhood caries intervention in Aboriginal Australian children: Follow-up at child age 9 years
Source: PLoS One. 2025 Sep 3;20(9):e0317024. doi: 10.1371/journal.pone.0317024 (PMC12407408; doi:10.1371/journal.pone.0317024)
Supplement: S7 Table — (DOCX) [file pone.0317024.s007.docx]

S7 Table: Models using IPCW for the mean number of DMFT at 9 years follow-up (RR, 95% CI)

|  | Model 1 | Model 2 | Model 3 | Model 4 |
| --- | --- | --- | --- | --- |
|  | RR (95% CI) | RR (95% CI) | RR (95% CI) | RR (95% CI) |
| **Intervention group** |  |  |  |  |
| DI | 0.89 (0.60-1.33) | 0.91 (0.61-1.37) | 0.57 (0.31-1.03) | 0.58 (0.31-1.08) |
| II | ref | ref | ref | ref |
| **Mothers’ characteristics at baseline** | |  |  |  |
| **Maternal age** |  |  |  |  |
| 14-24 | 1.28 (0.85-1.92) | 1.26 (0.82-1.93) |  | 1.40 (0.75-2.60) |
| 25+ | ref | ref |  | ref |
| **Education** |  |  |  |  |
| ≤12 years | *1.81 (1.09-3.04) | *1.77 (1.02-3.09) |  | **1.69 (1.07-3.97) |
| >12 years | ref | ref |  | ref |
| **Source of Income** |  |  |  |  |
| Centrelink | 0.88 (0.52-1.49) | 0.67 (0.36-1.24) |  | 0.94 (0.42-2.09) |
| Job | ref | ref |  | ref |
| **Residential location** |  |  |  |  |
| Non-metropolitan | 1.24 (0.81-1.89) | 1.26 (0.82-1.94) |  | 0.91 (0.50-1.66) |
| Metropolitan | ref | ref |  | ref |
| **Smoking status** |  |  |  |  |
| Current | 0.93 (0.59-1.46) | 0.92 (0.55-1.53) |  | 0.83 (0.41-1.71) |
| Former | *0.41 (0.21-0.80) | *0.41 (0.21-0.83) |  | 0.42 (0.17-1.02) |
| Never | ref | ref |  | ref |
| **Alcohol status** |  |  |  |  |
| Current | 0.77 (0.28-2.14) | 0.96 (0.34-2.76) |  | 0.88 (0.17-4.56) |
| Used | 1.09 (0.55-2.14) | 1.39 (0.69-2.81) |  | 1.12 (0.41-3.08) |
| Never | ref | ref |  | ref |
| **Children’s characteristics** |  |  |  |  |
| **Sex** |  |  |  |  |
| Male | 0.60 (0.40-0.90) |  | 0.71 (0.39-1.27) | 0.73 (0.40-1.34) |
| Female | ref |  | ref | ref |
| **Gestation** |  |  |  |  |
| Preterm | 2.22 (0.62-7.92) |  | 2.90 (0.44-19.22) | 3.41 (0.50-23.22) |
| Normal | ref |  | ref | ref |
| **Baby birth weight** |  |  |  |  |
| Low | 1.28 (0.49-3.35) |  | 0.79 (0.28-2.28) | 0.51 (0.16-1.60) |
| Normal | ref |  | ref | ref |
| **Breast feeding** |  |  |  |  |
| No | 1.32 (0.86-2.13) |  | 0.63 (0.35-1.12) | 0.56 (0.30-1.02) |
| Yes | ref |  | ref | ref |
| **Free sugar consumption of total energy intake** | |  |  |  |
| > 15% | 3.39 (0.57-20.33) |  | 2.25 (0.36-14.09) | 2.87 (0.45-18.46) |
| 11%-15% | 2.84 (0.52-15.66) |  | 1.80 (0.32-10.17) | 2.06 (0.35-12.01) |
| 5%-10% | 2.92 (0.51-16.69) |  | 1.72 (0.28-10.76) | 1.82 (0.28-11.76) |
| < 5% | ref |  | ref | ref |
| **Tooth brushing** |  |  |  |  |
| < 2/day | *1.82 (1.02-3.27) |  | 1.78 (0.82-3.89) | 1.76 (0.76-4.09) |
| ≥ 2/day | ref |  | ref | ref |

Notes: RR: risk ratio, IPCW: the inverse-probability-of censoring weighting, II: Immediate intervention, DI: delayed intervention; *P<0.05, **P<0.01, ***P<0.001.
