# Supplementary material for: Early childhood caries intervention in Aboriginal Australian children: Follow-up at child age 9 years
Source: PLoS One. 2025 Sep 3;20(9):e0317024. doi: 10.1371/journal.pone.0317024 (PMC12407408; doi:10.1371/journal.pone.0317024)
Supplement: S1 File — (DOCX) [file pone.0317024.s009.docx]

Inclusivity in global research

PLOS’ policy on inclusivity in global research aims to improve transparency in the reporting of research performed outside of researchers’ own country or community and ensures that PLOS publications reporting global research adhere to high standards for research ethics and authorship. Authors of relevant research articles may be asked to complete the questionnaire below, which outlines ethical, cultural, and scientific considerations specific to inclusivity in global research. This questionnaire may be requested when researchers have travelled to a different country to conduct research, if research uses samples collected in another country, research with Indigenous populations or their lands, or if research is on cultural artefacts. Researchers travelling to another country solely to use laboratory equipment will not normally be required to complete the questionnaire. However, the questionnaire can be requested at the journal’s discretion for any submission – if you have been requested to complete this questionnaire by the PLOS journal you submitted to, please do so.

Please complete the questionnaire below and include this as a Supporting Information file with your manuscript. Note that if your paper is accepted for publication, this checklist will be published with your article in the supporting information files. Please ensure that you reference the checklist in the main body of your manuscript. We suggest adding a subsection ‘Inclusivity in global research’ to your Methods section and adding the following sentence: “Additional information regarding the ethical, cultural, and scientific considerations specific to inclusivity in global research is included in the Supporting Information (SX Checklist)”

The questions have been designed to be applicable to a wide range of study types, and there are subsections for both human subjects research and non-human subjects research. If any of the questions are not relevant to your research please mark them as “N/A” as appropriate.

**Ethical considerations, permits and authorship**

*This section is applicable to all research types.*

Provide details as to who granted permissions and/or consent for the study to take place in the Methods section of your manuscript. This should include the names of **all** ethics boards, governmental organizations, community leaders or other bodies that provided approval for the study. If individuals provided approval refer to these people by their role or title but do not list their name(s).

Reported on page number: Pages 7 to 8

If there were any deviations from the study protocol after approval was obtained please provide details of these changes in the Methods section of your manuscript.
Did this study involve local collaborators that are residents of the country where the research was conducted or members of the community studied? If you do not have any authors from said communities, please provide an explanation for this below.

Reported on page number: N/A

Yes. Our study was conducted in collaboration with several communities and an Indigenous Reference Group (IRG), composed of respected Indigenous adults from across South Australia, who guided the research. Although not all individual members of the communities and the IRG are listed as authors, we have described their involvement and contributions within the study in the Methods section, acknowledged each community by name, and recognised their input in the Acknowledgements section.

Everyone listed as an author should meet PLOS’ criteria for authorship and all individuals who meet these criteria should be included in the author byline, rather than the acknowledgements. For further information please see the journal’s Authorship Policy.

**Human subjects research (e.g. health research, medical research, cross-cultural psychology)**

Did you obtain written informed consent from a representative of the local community or region before the research took place? How did you establish who speaks for the community? Details of written informed consent obtained from study participants should be reported separately in the Methods section of your manuscript.

1. **Yes, we obtained written informed consent from representatives of the local communities before commencing the research.**
2. **Professor Lisa Jamieson (Chief Investigator) sent an invitation letter to the communities. Once approval to participate was received, Ms Joanne Hedges (Chief Investigator and an Indigenous woman) held meetings with participants referred by the communities, during which she introduced the research aims and progress. All study participants provided written consent.**
3. **These procedures are detailed in the Methods section of our manuscript.**

How did members of the local community provide input on the aims of the research investigation, its methodology, and its anticipated outcome(s)?

**Members of the local community provided input on the aims, methodology, and anticipated outcomes of the research through ongoing consultation and collaboration, primarily facilitated by the Indigenous Reference Group (IRG).** The IRG, composed of respected Indigenous community members from across South Australia, played a key advisory role from the early stages of the project.

They contributed to refining the research questions to ensure cultural relevance and appropriateness, advised on ethical considerations, and guided the development of data collection methods to align with community values and expectations. Community feedback also helped shape the anticipated outcomes of the research, with an emphasis on producing findings that would be meaningful and beneficial to the communities involved.

When engaging with the local community, how did you ensure that the informed consent documents and other materials could be understood by local stakeholders?

**To ensure that the informed consent documents and other materials were clearly understood by local stakeholders, we employed a number of strategies.** All materials were written in plain, accessible language, avoiding technical or academic jargon. We worked closely with the Indigenous Reference Group (IRG) and community representatives to review and adapt documents for cultural appropriateness and clarity.

In addition, Ms **Joanne Hedges** (Chief Investigator and an Indigenous woman) met with participants face-to-face to explain the research aims, procedures, and their rights as participants, including the voluntary nature of participation and confidentiality safeguards. These discussions provided opportunities for questions and clarification, ensuring that informed consent was genuinely informed and freely given.

Will the findings of the research be made available in an understandable format to stakeholders in the community where the study was conducted (e.g. via a presentation, summary report, copies of publications, etc.)? Please provide details of how this will be achieved.

Yes, the study findings have been shared with the communities, for example, by providing summary reports and copies of the publication to the communities and interested participants.

**Non-human subjects research using specimens/ animals collected as part of the study, or those housed in archival collections. Examples include archaeology, paleontology, botany and zoology.**

Did the permission you obtained from a local authority to perform the study include an agreement on access to outputs and benefit sharing? This may include procedures to enable fair distribution of the benefits and resources arising from the research performed. Please include any details of Prior Informed Consent and Benefit Sharing Agreements obtained. These may be required by field-specific regulations, for example the Convention on Biological Diversity (CBD) and the associated Nagoya Protocol.

N/A

If the material used in your study was imported, please A) provide the year it was imported and B) indicate whether permits were obtained to import/export the materials used, C) provide details of any permits obtained. If this information is not available, please indicate this.

N/A

If you used archival specimens, please state how the material used in your study was acquired by the institute it is held in and provide details of any permits obtained for the original excavations/ sample collection. If this information is not available, please indicate this.

N/A

How was the potential cultural significance of the materials collected in your study to local communities considered in your research design? Were Indigenous peoples and/or local researchers and institutions involved with archaeological excavations / collection of specimens? If so, please provide a description of their involvement.

Our study was developed in partnership with local Aboriginal communities and endorsed by the study’s Aboriginal Reference Group. Consultation with the South Australian Aboriginal community was done through 3 focus group discussions (one metropolitan, two regional) and via individual consultation with members of the study’s Aboriginal Reference Group.

If your manuscript includes photographs of human remains please indicate whether authors obtained permission from descendants or affiliated cultural communities to do so.

No, our manuscript did not include photographs of humans.
