# Supplementary material for: Early childhood caries intervention in Aboriginal Australian children: Follow-up at child age 9 years
Source: PLoS One. 2025 Sep 3;20(9):e0317024. doi: 10.1371/journal.pone.0317024 (PMC12407408; doi:10.1371/journal.pone.0317024)
Supplement: Attachment — Submitted filename: Response to reviewers_R1.docx [file pone.0317024.s011.docx]

**Response to reviewers**

**Reviewer #1**:

**The study is interesting, but several aspects require clarification.**
**1. How exactly was "sugar-sweetened beverage (SSB) consumption as a percentage of total food intake" or "percentage of free sugar consumption as a proportion of total energy intake" assessed? Which of these variables was included in the analysis?**

1) The percentage of total energy intake from free sugar was calculated using dietary data collected at the 2-year follow-up, based on the average of one 24-hour dietary recall conducted by a trained researcher using a food model booklet, and, where available, up to two additional 24-hour diet diaries. We have provided further details on how this was assessed (Line 167-171, page 9). This variable was included in the analysis of dmft for primary dentition (Line 191-193, page 10)
2) The proportion of sugar-sweetened beverage (SSB) consumption relative to total food intake was derived from dietary data collected at the 7-year follow-up (Line 179-182, page 9) and was included in the DMFT analysis for permanent dentition (Line 191- 192, page 10).

**2. On what basis was alcohol consumption categorised for women? What threshold was used to assign them an alcohol consumption status?**

Alcohol consumption was identified from responses to ‘What is your Alcohol drinking status?’ Then it was categorised as 1) Currently drink alcohol. 2) Used to drink alcohol, 3) Have never drunk alcohol. We have added this information in the Methods section (Line 175-178, Page 9).

**3. Why do the authors conclude that their study indicates that stopping smoking during pregnancy can reduce the incidence of ECC? The evidence for this statement is unclear.**

Many thanks. We have deleted this unclear sentence (Line 408, page 24).

**4. The following sentence is too long and unclear:
"It cannot be denied that the prevalence of missing teeth due to dental caries in the primary dentition was much higher due to early extractions or exfoliation, resulting in premature eruption of permanent teeth, which increased the risk of dental caries in the permanent dentition."**

**Could the authors clarify this statement? Additionally, why do they suggest that the prevalence of missing teeth due to dental caries is higher due to exfoliation? Naturally exfoliated teeth are not included in the caries index, and severely carious teeth often do not exfoliate naturally.**

Thanks. We have rewritten the sentence as ‘The high prevalence of missing teeth in the primary dentition—primarily due to early extractions from severe caries rather than natural exfoliation—contributed to the premature eruption of permanent teeth, thereby increasing the risk of dental caries in the permanent dentition (Line 354-357, Page 21-22).

5. **Based on the results of the current study, it is not possible to conclude that "an ECC intervention delivered in early childhood had a long-term effect on reducing dental caries experience in the primary dentition at child age 9 years." There was no control group without intervention, and a comparison with national data alone is insufficient to confirm the intervention's success.**

Thank you for your comments. Although we cannot definitively conclude that early childhood caries (ECC) intervention has a long-term effect on reducing dental caries experience, the data suggest a potential benefit. Specifically, the mean dmft was lower in the immediate intervention (II) group (3.20) compared to the delayed intervention (DI) group (3.61) at 9 years (S1_Table), indicating a possible positive impact of early intervention.

We have rewritten the sentence as ‘The present study suggests that an early childhood ECC intervention may have a positive impact on reducing dental caries experience in the primary dentition by age 9 years’ (Line 412-413, Page 24, and Abstract).

**Reviewer #2:**

**This paper is a very valuable report on the effectiveness of early intervention in preventing dental caries in indigenous Australian children up to the age of 9 years.
However, the following points require reconsideration.**

1. **The authors have already reported results up to 5 years of age in another journal (ref. 18), and more consideration should be given to the effect on caries in the permanent dentition.**

Thanks, we have added more consideration in the Discussion section (Line 361-362, Page 22).

2. **Why is early permanent tooth caries more common in group II?**

Early permanent tooth caries is more common in Group II, possibly because early intervention was no longer effective by the time the permanent teeth erupted. Additionally, the DI group received the same Early Childhood Caries (ECC) intervention after a 2-year follow-up, which may have provided more opportunities for preventive measures to influence the development of caries in permanent teeth.

3. **Why is there more caries in boys in the deciduous dentition but more in girls in the permanent dentition?**

This pattern may be explained by gene-by-sex interactions influencing caries experience in both primary and permanent dentitions (ref #1): ‘*In the primary dentition, the magnitude of the effect of genes was greater in males than females. In the permanent dentition, different genes may play important roles in each of the sexes’*.

Also, our findings align with those of a previous study (ref #2), which reported that males had a higher overall mean dmft and a greater number of untreated decayed primary teeth, while females exhibited higher DMFT scores in the permanent dentition (See S1_Table).

*Reference*

1. *Shaffer JR et al. Genetic Susceptibility to Dental Caries Differs between the Sexes: A Family-based Study. 2016; 49 (2): 133-140.*
2. *Obradovic M et al. Caries Experience in Primary and Permanent Dentition in Children Up to 15 Years of Age from Bosnia and Herzegovina—A Retrospective Study. Child. 2023; 10 (4): 754.*
4. **Need to explain Model in Tables 2 and 3.**

Thanks. We have added footnotes to explain the Models in Tables 2, 3, 5 and 6.
